# Tracking the Prevalence of Obesity in Portuguese School-Aged Children: What Future to Expect?

**DOI:** 10.3390/children11080976

**Published:** 2024-08-13

**Authors:** Nelson Valente, Pedro Forte, José E. Teixeira, Pedro Afonso, Sérgio Ferreira, Daniel A. Marinho, Pedro Duarte Mendes, Ricardo Ferraz, Luís Branquinho

**Affiliations:** 1 Biosciences Higher School of Elvas, Polytechnic Institute of Portalegre, 7300-110 Portalegre, Portugal; nelsonvalente@ipportalegre.pt (N.V.); luisbranquinho@ipportalegre.pt (L.B.); 2 Research Center of Higher Institute of Educational Sciences (CI-ISCE), 4560-547 Penafiel, Portugal; pedromiguel.forte@iscedouro.pt; 3 Life Quality Research Centre (CIEQV), 2040-413 Portalegre, Portugal; 4 Department of Sports Sciences, Polytechnic Institute of Bragança, 5300-252 Bragança, Portugal; jose.eduardo@ipg.pt; 5 Research Center in Sports Sciences, Health Sciences and Human Development (CIDESD), 6201-001 Covilhã, Portugal; dmarinho@ubi.pt (D.A.M.); ricardo.ferraz@ubi.pt (R.F.); 6 LiveWell—Research Centre for Active Living and Wellbeing, Polytechnic Institute of Bragança, 5300-252 Bragança, Portugal; 7 Department of Sports Sciences, Higher Institute of Educational Sciences of the Douro, 4560-708 Penafiel, Portugal; sergioferreira98@hotmail.com; 8 Department of Sports Sciences, Polytechnic of Guarda, 6300-559 Guarda, Portugal; 9 SPRINT—Sport Physical Activity and Health Research & Inovation Center, 2040-413 Rio Maior, Portugal; 10 Department of Sports Sciences, University of Trás-os-Montes e Alto Douro, 5000-801 Vila Real, Portugal; pmvafonso@gmail.com; 11 Department of Sports Sciences, University of Beira Interior, 6201-001 Covilhã, Portugal; 12 Sport, Health & Exercise Research Unit (SHERU), Polytechnic Institute of Castelo Branco, 6000-084 Castelo Branco, Portugal

**Keywords:** childhood, cardiovascular health, body composition

## Abstract

Background: Childhood obesity presents a significant public health concern globally, with implications for cardiovascular health and metabolic syndrome. In Portugal, approximately 31.6% of children are affected, highlighting the urgency for intervention strategies. This study aimed to assess the prevalence of overweight and obesity in Portuguese school-aged children, with a focus on sex and age differences. Methods: Anthropometric measurements were conducted on 1564 children aged 6–10 years, including weight, height, and skinfold thickness. Body Mass Index (BMI) and the percentage of body fat were calculated using established methods. Results: The results revealed significant differences in BMI (≤0.001) and body fat percentage (≤0.001) among different BMI categories, with a notable prevalence of overweight and obesity, particularly among boys. A total of 37% of the studied population is overweight or obese, among which 40.1% and 33.9% are boys and girls, respectively. Conclusions: This study highlights statistically significant differences in BMI and body fat percentage for both sexes in different BMI categories. A large proportion of the population is overweight or obese, with a greater prevalence in boys. In short, childhood obesity has a negative impact on body composition and is associated with significant differences in anthropometric parameters, emphasizing the importance of preventative and intervention strategies to address this health problem.

## 1. Introduction

Childhood obesity is a significant public health concern globally, with studies indicating its increasing prevalence and adverse health effects [1]. Research has shown that childhood obesity can result in cardiovascular and endocrine dysregulation, insulin resistance, and metabolic syndrome, even without concurrent conditions like hypertension and type 2 diabetes [2]. In Portugal, studies have revealed a high prevalence of overweight and obesity among children, affecting approximately 31.6% of the child population [3]. The impact of childhood obesity on body composition extends beyond aesthetics, affecting various physiological and metabolic processes that influence overall health [4]. Excess adipose tissue not only impacts physical appearance but also contributes to health issues such as type 2 diabetes, metabolic syndrome, inflammation, dyslipidemia, and cardiovascular diseases [5]. Additionally, childhood obesity is a significant risk factor for adult obesity, with around 50% of obese children likely to remain obese into adulthood [4]. Efforts to combat childhood obesity have explored interventions like lifestyle modifications and the involvement of community health workers [6,7]. Lifestyle interventions are crucial in addressing childhood obesity, aiming to mitigate the physiological and psychological consequences associated with this epidemic [7]. Community health workers play a vital role in reducing disparities in childhood obesity by leveraging their close connections to the communities they serve [6].

The etiology of obesity is a complex issue influenced by genetic predispositions, environmental factors, and lifestyle behaviors. Recent research has highlighted the significant role of decreased physical activity, poor dietary habits, and sedentary behavior in the development of obesity [8,9]. Sedentary behavior has been identified as a key factor leading to an energy imbalance and subsequent calorie storage in adipose tissue [10]. Studies have demonstrated that individuals who are overweight during childhood are at a higher risk of developing obesity in adulthood, especially if the condition persists into adolescence [11]. Healthcare providers are increasingly faced with the challenge of managing diseases like obesity and type 2 diabetes in children, conditions that were traditionally associated with adults [12]. While there have been reports indicating a slight decrease in the prevalence of childhood obesity, it remains a significant public health concern requiring effective prevention and intervention strategies [13,14]. Family-based interventions have emerged as a crucial strategy in preventing childhood obesity, recognizing the influence parents have on children’s energy-balance behaviors [15]. Recent literature has underscored the importance of understanding the interactions between various risk factors for obesity, such as sedentary behavior, dietary patterns, and genetic predisposition [16]. Addressing these factors through tailored interventions and policy initiatives is essential in combating the obesity epidemic and its associated health risks.

The assessment and classification of childhood obesity involves a range of methodologies and criteria that have evolved with advancements in research on human body composition. Recent studies have emphasized the importance of understanding fat distribution and fat-free mass (FFM) through multi-compartmental models [17]. While molecular multi-compartmental models offer accuracy, practical considerations have led to the adoption of more feasible methods such as anthropometry, bioelectrical impedance, air displacement plethysmography, and dual-energy X-ray absorptiometry (DXA) for assessing body composition [18]. Body Mass Index (BMI) has been extensively used in classifying obesity in adults, but its application in children has been a topic of debate. The Centers for Disease Control and Prevention (CDC) in the US established specific criteria in 2000 for children, designating those between the 85th and 95th percentiles as overweight and those above the 95th percentile as obese [19]. Studies have demonstrated correlations between childhood BMI values and adiposity in adulthood, supporting its role as a screening tool [20]. However, BMI’s limitation in distinguishing between fat mass and FFM underscores the need to estimate these components for a more comprehensive assessment of body composition [21]. The distribution of adiposity, particularly around the trunk, has garnered attention due to its implications for metabolic health. Waist circumference has been utilized as a practical indicator of adipose tissue distribution and has been associated with the risk of cardiovascular disease, especially in adults [22]. Despite BMI’s simplicity and association with basic anthropometric data like weight and height, efforts to develop more precise methods for evaluating body composition in children persist as a significant challenge [23].

This article aims to evaluate the prevalence of overweight and obesity in Portuguese school-aged children, with a focus on sex and age differences, as well as changes in lean mass by sex and age. Additionally, it will address the consequences of childhood obesity on physical development, metabolic function, and cardiovascular health in children. It is hypothesized that there are significant differences between sexes and ages.

## 2. Materials and Methods

### 2.1. Participants

The sample consisted of 1564 children, 792 males and 772 females, of school age (6–10 years) in the 1st cycle of Portuguese basic education. All participants underwent the assessments that were part of the study after obtaining informed consent from each parent or guardian of the children, who attested that they had been adequately informed about the objectives, potential advantages, and risks of the assessments to be carried out. The study was conducted in accordance with the Declaration of Helsinki and approved by the Institutional Scientific Board of ISCE Douro (PF:10.2021, approved in October 2021) for studies involving humans.

### 2.2. Research Design

A cross-sectional, observational, and prospective study was conducted. The inclusion criteria for this research were as follows: (1) the study included children aged 6 to 10 years who were enrolled in the 1st cycle of Portuguese basic education; (2) all anthropometric measurements were performed in the morning, and the children had to be present for assessments during that time; (3) all participants required informed consent from a parent or guardian, indicating that they were adequately informed of the study objectives and potential benefits and risks. In contrast, the exclusion criteria for this study were (1) children with medical conditions that could affect anthropometric measurements, such as mobility impairments or chronic illnesses that affect growth and development, were excluded from the study, as were children if they or their guardians did not give informed consent; (2) children were also excluded from the final analysis if they were not present at the scheduled assessment times or if they had incomplete data.

### 2.3. Anthropometric Assessment

The anthropometric measurements were assessed only in the morning. Weight, height, and skinfold thickness (triceps and subscapular) were evaluated. Body Mass Index (BMI) was calculated using Quetelet’s formula, dividing weight (kg) by height (m) squared and classified according to WHO standards [24]. To determine the percentage of body fat, subcutaneous triceps and subscapular skinfolds were measured, and values were calculated using the equations previously reported in the literature [25]. Skinfold measurements were taken on the right side, with skin and adjacent subcutaneous tissue being pinched. To pinch the skinfold, two fingers were positioned 8 cm apart (using the length of the index finger as a reference). The thumb and index finger were 1 cm away from the measurement site, and approximately 3 s were provided before taking the reading on the Adipometer. Two measurements were taken at each anthropometric site. If the difference between the two measurements exceeded 1 mm, a new measurement was taken, and subsequently, the average was calculated. Alternate measurements were taken to allow for skin thickness and texture recovery. All skinfolds were measured to the nearest 0.5 mm [26]. Two observers performed the measurement three times and the average was used for further analysis. The intra-class correlation coefficient (ICC) showed almost perfect agreement (ICC = 0.88 to 0.92) for intra- and inter-observer reliability.

**Figure 1:**
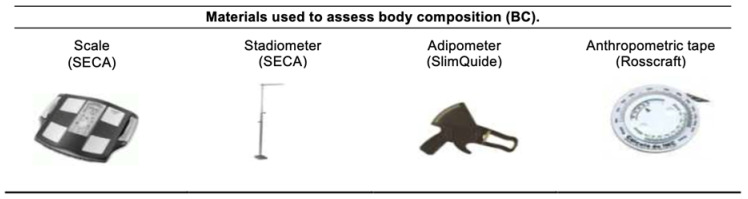
Materials used to assess body composition (BC).

For the collection of anthropometric data, a Rooscraft anthropometric kit (Rosscraft, Vancouver, Canada) was used. Weight and height were measured with a SECA brand scale and stadiometer (SECA, Hamburg, Germany) with a margin of error of 100 g. Abdominal circumference was measured using a Rosscraft brand tape measure (Rosscraft, Canada) with a margin of error of 0.1 cm. Triceps and subscapular skinfolds were measured using a Slim Guide caliper (Creative Health Products, Ann Arbor, MI, USA). Before conducting the tests, a clarification session was held with the students regarding the study objectives and assessment procedures. Figure 1 and Table 1 show the materials used for the collection of anthropometric data and the variables resulting from this collection.

### 2.4. Statistical Analysis

The initial exploratory data analysis was conducted in two main stages. Firstly, potential missing cases and data entry errors for all considered variables were identified using descriptive tables. Subsequently, a graphical analysis was performed to identify possible outliers, employing boxplots. To assess the normality of the sample, Tukey’s test was utilized. For variables that did not exhibit a normal distribution, logarithm transformation was applied. Even after this procedure, variables that remained non-normally distributed were retained in the statistical analysis. This decision was based on the central limit theorem, which states that with an increasing sample size (*n* ≥ 30), the distribution of sample means approaches normality, thus reflecting the population mean [28].

In the descriptive analysis, the mean was used as a measure of central tendency, and the standard deviation was employed as a measure of variability. In the inferential statistical stage, an analysis of variance (ANOVA One-Way) was applied to compare the means among BMI categories, along with post hoc tests to identify significant differences between BMI categories for both boys and girls. To compare differences between genders across different ages and BMI categories, an independent sample *t*-test was employed. In the analysis of associations between variables, an exploration of correlation matrices was conducted, utilizing Pearson correlation coefficient (r) for variables that met the assumptions of normality. The significance level was maintained at 5% for all statistical calculations. All calculations were performed using the SPSS statistical package version 24.0 for Windows.

## 3. Results

Table 2 displays the descriptive values of means, standard deviations, minimums, and maximums of the anthropometric variables in the assessment of body composition. The results are presented for the entire sample by gender and different age groups. There is a trend of increasing mean values with age for all evaluated parameters. Boys and girls presented very similar mean values across different age groups in all evaluated parameters. The anthropometric data reveal that both male and female children exhibited an increase in weight, height, BMI, triceps, and subscapular skinfold thickness with age. Notably, boys consistently had higher average BMIs and fat mass percentages compared to girls across all age groups, with a marked increase in these measures from age 6 to 10. Regarding Body Mass Index (BMI), the mean values, although close, were higher in males, as is the case for the percentage of body mass fat (%BF), except at nine years old.

Table 3 reports the mean values, standard deviations, minimums, maximums, and analysis of variance of the means for the anthropometric variables under study across different BMI categories and for both genders. The analysis of variance across BMI groups revealed that there were notable differences in several anthropometric variables between boys and girls (F = 22.43 to 844.36; *p* < 0.001). There was a notable increase in BMI, weight, height, tricipital and subscapular skinfold thickness, and body fat percentage as BMI categories rose from underweight to obese. Compared to children in lower BMI groups, children in the obese category exhibited the highest mean values across all of these parameters. Waist circumference also demonstrated a gradual increase with a higher BMI, indicating a potential for greater central adiposity in children with excess weight.

The descriptive values of BMI indicate a trend towards higher mean values in females across all BMI categories, except for the underweight (UW) category. However, for the percentage of body fat (%BF) and waist circumference (WC), higher mean values were observed in males, except in the overweight (OW) category. Statistically significant differences (*p* ≤ 0.05) were observed for all analyzed anthropometric parameters (except for age) in the analysis of variance between the mean values across different BMI categories and for both genders.

Table 4 indicates that the study sample does not exhibit statistically significant differences between BMI categories for the variable age in both genders, except between the normal weight (NW) and overweight (OW) categories for females. The observed difference may be related to the variance in the number classified with NW and OW among females. However, there is a uniform distribution of mean ages across different BMI categories in both genders for the remaining comparisons. With the exception of a little variation in girls’ BMI categories between normal and excess weight (*p* = 0.032), the study did not find any significant age differences across BMI categories for either gender. The average age differences between these groups had narrow confidence intervals, suggesting little variance. Overall, the findings imply that the age distribution of the participants was not substantially impacted by BMI categories.

**Figure 2:**
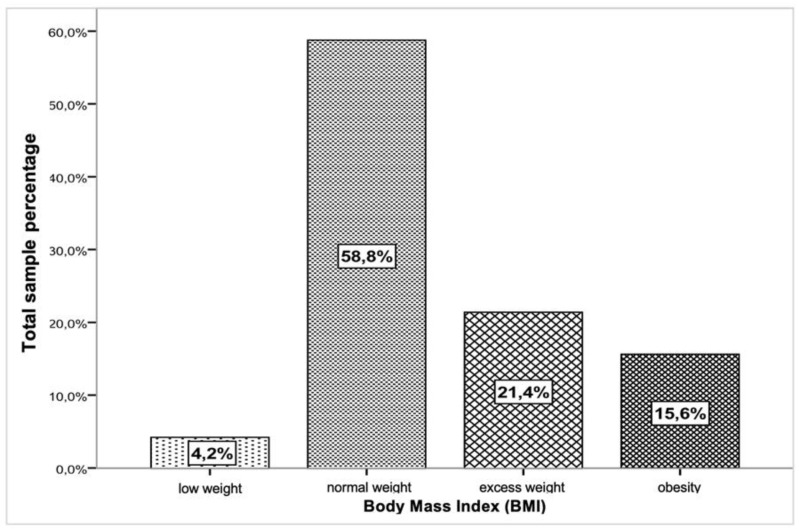
Percentage distribution of the entire sample by BMI categories (*n* = 1564).

Figure 2 illustrates the percentage distribution of the entire sample across different BMI categories.

**Figure 3:**
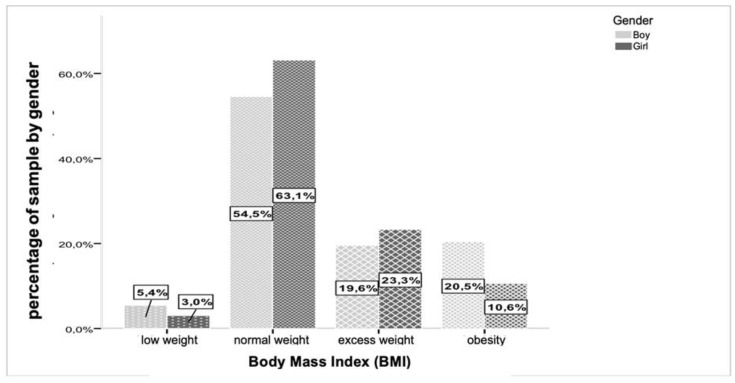
Percentage distribution of the entire sample, by gender and BMI categories (total *n* = 1564).

Through Figure 2, it is observed that 37% of the studied population has excess weight, is overweight, or obese (OB). Figure 3 reflects the percentage distribution of BMI categories by gender, showing percentage values with greater relevance of excess weight (overweight and obesity) for males (40.1%) and 33.9% for females.

**Figure 4:**
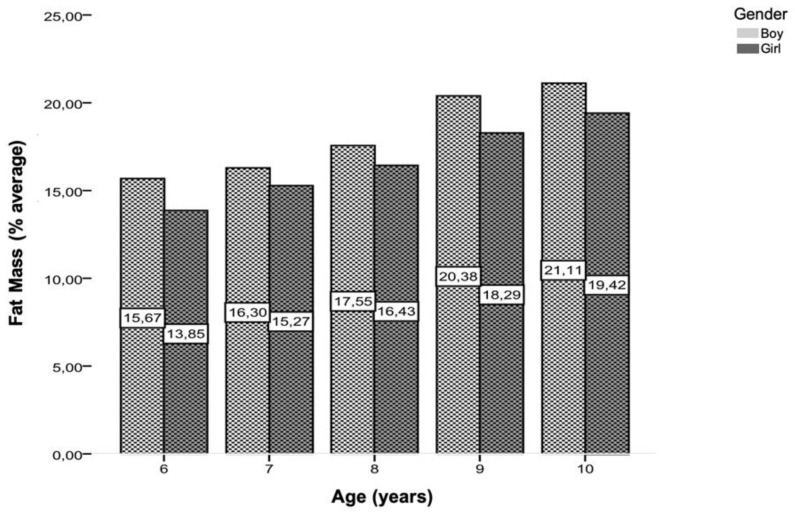
Distribution of the average percentages of MG for the entire studied population, by gender and age.

Figure 4 graphically represents the evolution of the % BF value across different ages and genders. An increase is observed with advancing age, with a higher value for all ages in males.

Table 5 and Table 6 refer to the multiple comparisons between BMI categories by gender in the anthropometric variables relevant to body composition assessments.

It is observed (Table 5) through post hoc testing that all variables presented statistical significance (*p* ≤ 0.05) in the multiple comparisons between different BMI categories in males.

**Figure 5:**
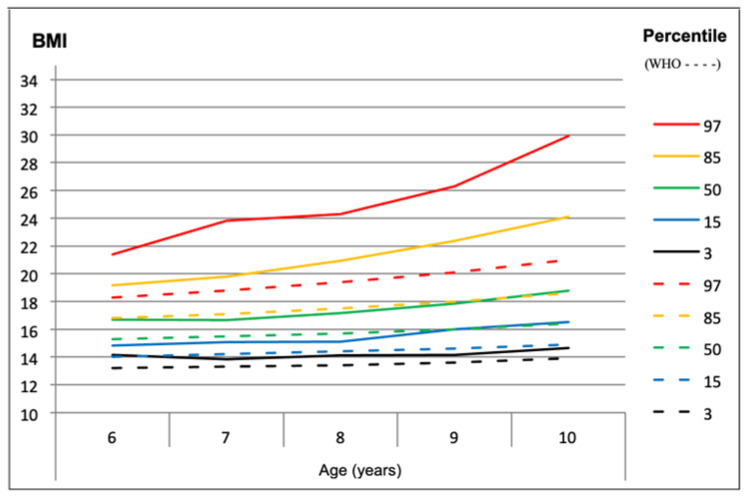
Curves of the BMI percentile values of the study sample compared to the WHO percentile values, for males at different ages (*n* = 792).

**Figure 6:**
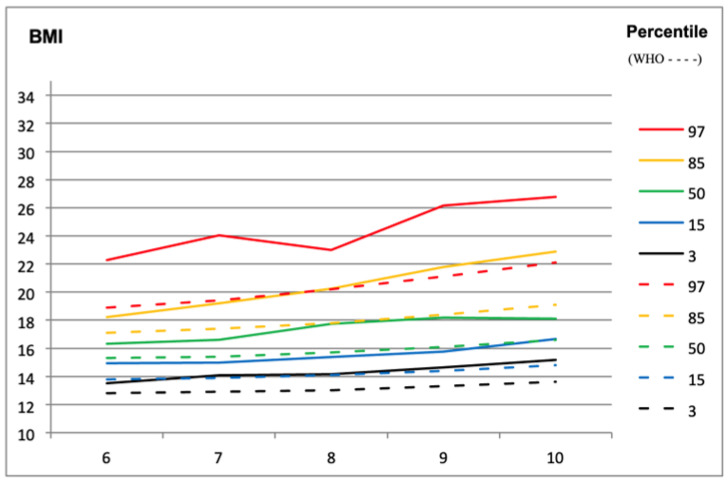
Curves of the BMI percentile values of the study sample compared to the WHO percentile values, for females at different ages (*n* = 772).

Through Figure 5 and Figure 6, information about the development of body weight can be observed to establish a reference based on the 2007 WHO curves in the children in our sample within the respective age group. The results indicate that for all percentage values and in both sexes, there are BMI values higher than the reference values of the WHO.

## 4. Discussion

The objective of this study was to assess the prevalence of overweight and obesity in Portuguese school-aged children, with a focus on sex and age differences, as well as changes in lean mass by sex and age. Significant differences in BMI and body fat percentage, with a notable prevalence of overweight and obesity, were verified in the sampled children, particularly among boys. The results support the hypothesis that there are significant differences between sexes and ages.

Current research identified that children aged 6–9 years have been identified as having a significantly higher likelihood of obesity in adulthood, highlighting the urgency of addressing obesity at a young age to curb its long-term impact [29]. In a study conducted by the Harvard Growth Study, which followed 1857 school-age subjects over 8 years, findings revealed that overweight children had a higher overall risk of mortality and were twice as likely to develop heart disease compared to leaner counterparts [30]. Bar-Or et al. [27] further highlighted that approximately 40% of obese children and 70% of obese adolescents are at risk of becoming obese adults [31]. Children and adolescents are particularly vulnerable to a spectrum of morbidities in adulthood, including type 2 diabetes, hypertension, cardiovascular diseases, knee osteoarthritis, and various physical and psychological irregularities [32]. Portugal, among other developed European countries, exhibits one of the highest prevalences of overweight and obese children [33]. Recent literature has underscored the critical importance of comprehending the long-term implications of childhood obesity on adult health outcomes. Studies have consistently shown that childhood obesity significantly elevates the risk of developing chronic conditions in adulthood, underscoring the urgent need for early intervention and prevention strategies [34]. The global prevalence of childhood obesity has been a mounting concern, with various countries reporting alarming rates of overweight and obesity among children and adolescents [35]. In our study, which followed 1857 school-age subjects over 8 years, it was observed that BMI values increased with age, with values at 6 years old and at 10 years old reflecting the natural growth in height and weight parameters [36,37]. The results from our study revealed a spectrum of BMI classifications, with 58.8% classified as normal weight. Notably, the incidence of overweight (21.4%) and obesity (15.6%) in our sample indicated a higher prevalence, with 37% of the evaluated child population falling into the overweight or obese categories, surpassing previous findings [38,39,40,41]. When analyzing the data by gender, the prevalence of overweight and obesity was more pronounced. In males, there was a prevalence of 40.1% for overweight and obesity combined, with 20.5% classified as obese. In females, the prevalence of obesity decreased to 10.6%, while the prevalence of overweight increased to 23.3% [42,43]. Furthermore, our analysis did not reveal statistically significant differences in BMI categories with age for both genders, except for females between normal weight and overweight categories, indicating an increase in body composition components with age [44,45]. Recent studies have emphasized the need for early intervention strategies to address the long-term health implications associated with childhood obesity, including the increased risk of chronic conditions in adulthood [46,47]. The prevalence of childhood obesity remains a critical public health issue, necessitating comprehensive approaches to combat this growing epidemic and promote better health outcomes for children and adolescents [48,49].

Another study conducted in Portugal, focusing on children aged between 3 and 5 years, revealed concerning rates of overweight and obesity. The study found that 19.1% of girls and 15.9% of boys were overweight, while 15.3% of girls and 13.9% of boys were classified as obese. Subsequent research in the same municipality, tracking 41 preschool-aged children between 2005 and 2008, showed a notable increase in overweight percentages, with figures rising from 7.14% in 2005 to 28.57% in 2008 for girls and from 7.41% in 2005 to 11.11% in 2008 for boys [50]. Incorporating percentile curves from the current study and taking into account the reference for comparison with WHO curves from 2007, the variation in percentile BMI values in the sample indicated higher values between 7 and 9 years, potentially due to the larger sample size in this age range compared to 10 years. Comparing the curves from the sample to reference curves and standard deviations suggested a manifestation of overweight cases, with higher percentile values observed in the sample, particularly from 8 years onwards, indicating an increase in overweight and obesity cases [51]. The discrepancy between the sample’s BMI values and the reference curves raises questions about the appropriateness of using WHO curves from 2007 for the Portuguese population. There is a hypothesis suggesting that Portuguese children may have specific genetic characteristics leading to a different weight/height relationship compared to the reference population, potentially resulting in inaccuracies in BMI classification [52]. These findings underscore the importance of considering genetic and environmental factors in childhood obesity research. Recent studies have highlighted the complex interplay between genetic predisposition, lifestyle habits, and gene–environment interactions in the development of childhood obesity [53,54]. Understanding these factors is crucial for developing effective prevention and intervention strategies to address the rising prevalence of childhood obesity and promote better health outcomes for children and adolescents. While BMI data alone may not provide a comprehensive understanding of body composition, it is reasonable to suggest that individuals with increasing BMI values may also experience a rise in body fat percentage (%BF) in the studied population. The mean values of %BF and waist circumference were slightly higher in males, except in the overweight category. At 6 years old and at 10 years old, higher %BF values were noted, particularly in males. Similarly, higher waist circumference values were observed at 6 years and at 10 years, reflecting the natural anthropometric growth of children.

Comparisons among different BMI categories in both boys and girls revealed significant differences in the BMI, %BF, and waist circumference components, confirming an increase in mean values with the severity of BMI. Children tend to accumulate more subcutaneous fat in the extremities than in the trunk up to 5 or 6 years of age. Subsequently, they also accumulate subcutaneous fat in the upper body, indicating a shift in fat distribution as they age. The discrepancy between the sample’s BMI values and reference curves raises questions about the appropriateness of using standard WHO curves for the studied population. It is hypothesized that specific genetic characteristics in Portuguese children may lead to variations in weight/height relationships, potentially affecting BMI classification accuracy. Understanding these nuances in body composition assessment is crucial for tailored interventions and accurate health evaluations in children.

The present study is subject to several limitations that should be considered when interpreting its findings. Firstly, the sample may restrict the generalizability of the results to broader populations. Additionally, the cross-sectional design of the study limits its ability to establish causal relationships between variables, thereby impeding the inference of temporal associations. Lastly, the study may have overlooked important factors, such as socioeconomic status, cultural differences, and lifestyle behaviors, as well as external influences, which could confound the interpretation of the findings.

## 5. Conclusions

This study showed statistically significant differences in the BMI and body fat percentage for both sexes across different BMI categories. A total of 37% of the studied population is overweight or obese, among which 40.1% and 33.9% are boys and girls, respectively. In summary, childhood obesity negatively impacts body composition and is associated with significant differences in anthropometric parameters, emphasizing the importance of preventive and intervention strategies to address this health issue.

## Figures and Tables

**Table 1:** Anthropometric measurements.

	Anthropometric Measurements
**Weight**	After checking the scale, the children stood in the center of the platform with their weight distributed over both feet and looking straight ahead in the anthropometric position. At the time of measurement, the children were dressed only in shorts or bathing suits.
**Height**	It was measured between the vertex (highest point of the skull when the head is held in a position known as the “Frankfort Plane”, [27]) and the ground reference plane. They were barefoot, wearing shorts or a swimsuit at the time of measurement and in an anthropometric position.
**WC**	Measured just above the iliac crests.
**Triceps grip**	Halfway (posterior) between the external projection of the acromion and the olecranon. Vertical pleat.
**Subscapular fold**	Find the innermost and lowest edge of the shoulder blade and mark the point. Mark the oblique line over the skin’s own natural cleavage line. The point will serve as a guide for marking the vertical and horizontal line over the cleavage line. The pleat is highlighted obliquely at an inclination of ±45°.

**Table 2:** Descriptive values of the anthropometric assessment of the entire sample (total *n* = 1564; ♂ *n* = 792, ♀ *n* = 772).

Variables		Age (Years)				
		6 y (♂ *n* = 146 ♀ *n* = 138)	7 y (♂ *n* = 195 ♀ *n* = 223)	8 y (♂ *n* = 198 ♀ *n* = 179)	9 y (♂ *n* = 186 ♀ *n* = 171)	10 y (♂ *n* = 67 ♀ *n* = 61)
**Weight (kg)**	♂	24.70 ± 4.51 17.60–39	24.43 ± 5.56 18–46.59	30.86 ± 7 19.80–65.80	35.37 ± 8.17 19.50–72.60	38.05 ± 8.76 24–66
	♀	24 ± 3.19 17–36	27.17 ± 5.34 18.50–48.90	30.28 ± 5.57 19–52	35.05 ± 7.55 20.80–62	39.75 ± 9.71 25.30–69.80
**Height (cm)**	♂	120.36 ± 5.56 107.3–136	125.37 ± 6.18 108–145	130.89 ± 6.04 116–150.30	136.26 ± 6.09 120–153	139.06 ± 6.49 126.80–155
	♀	119.96 ± 5.59 106–149	125.47 ± 6.18 110–168	129.96 ± 5.39 116.40–145	136.32 ± 6.47 118.20–155	142.73 ± 7.52 128–163.70
**BMI (kg·m^−2^)**	♂	16.91 ± 2.01 9.88–23.89	17.32 ± 2.47 12.27–27.10	17.86 ± 2.85 13.11–29.24	18.89 ± 3.28 3.04–31.01	19.55 ± 3.71 14.08–32.41
	♀	16.63 ± 2.02 12.84–25.42	17.15 ± 2.46 13.06–26.34	17.83 ± 2.43 12.36–26.53	18.75 ± 3.12 13.79–29.49	19.29 ± 3.07 14.97–27.89
**Tricipital fold** **(mm)**	♂	10.77 ± 3.46 4–21.50	11.20 ± 4.52 4–24.50	11.94 ± 5.20 4–27	14.27 ± 6.24 5–39	14.82 ± 5.96 6–31
	♀	9.65 ± 3.91 4–22	10.71 ± 4.88 4–31.50	11.46 ± 4.99 4.50–31	13.20 ± 6.19 4.50–35	14.02 ± 5.85 6–28
**Subscapular** **fold** **(mm)**	♂	5.52 ± 2.20 2–14	6.07 ± 3.84 2.50–23	6.83 ± 4.19 2–24	7.94 ± 4.73 3–29.50	8.23 ± 5.08 3.50–25.50
	♀	5.16 ± 2.58 2–16	6 ± 3.96 2–29.50	6.57 ± 3.99 2.50–30.50	7.40 ± 4.71 2.50–29	7.98 ± 5.28 2.50–27
**Add pleats** **(mm)**	♂	16.29 ± 5.31 7–32.50	17.27 ± 7.82 7–46.25	18.76 ± 8.77 7–49	20.60 ± 10.52 7.50–64	23.05 ± 10.47 10.50–49.50
	♀	14.80 ± 6.29 6.50–37.50	16.67 ± 8.49 6.50–61	18.02 ± 8.60 7.50–57	23.05 ± 10.47 10.50–49.50	22 ± 10.52 9–52
**WC (cm)**	♂	56.68 ± 4.87 48.20–71.90	57.85 ± 6.88 47–85.50	60.02 ± 7.43 43–81.70	62.94 ± 8.92 49–87	62.69 ± 7.30 53.90–82
	♀	53.81 ± 4.80 46–69	57.32 ± 6.81 45.30–85.70	58.57 ± 6.53 47.50–80.50	62.69 ± 7.30 53.90–82	61.62 ± 10.05 34.70–77.80
**BF (%)**	♂	15.67 ± 4.82 6.38–29.18	16.30 ± 6.54 6.38–37.8	17.55 ± 7.20 6.38–39.97	18.29 ± 7.57 6.74–44.64	21.11 ± 8.01 10.12–40.36
	♀	13.85 ± 5.26 5.60–30.45	15.27 ± 6.49 5.60–43.01	16.43 ± 6.58 6.74–40.82	21.11 ± 8.01 10.12–40.36	19.42 ± 7.48 8.42–38.09

Note: Gender (masculine ♂ and feminine ♀); Mean and Standard Deviation (m ± SD); Minimum and Maximum (min.–max.). Abbreviations: BF%—percentage of body fat mass; BMI—Body mass index; WC—Waist perimeter; y—years.

**Table 3:** Descriptive values and analysis of variance of means between BMI categories.

		BMI Categories					
		Low Weight ♂ *n* = 43 ♀ *n* = 23	Normal Weight ♂ *n* = 432 ♀ *n* = 487	Excess Weight ♂ *n* = 155 ♀ *n* = 180	Obesity ♂ *n* = 162 ♀ *n* = 82	*F*	*p*
**Age (years)**	♂	7.86 ± 1.17 6–10	7.75 ± 1.24 6–10	7.74 ± 1.21 6–10	7.93 ± 1.24 6–10	1.04	0.375
	♀	7.74 ± 1.14 6–10	7.66 ± 1.22 6–10	7.89 ± 1.21 6–10	7.82 ± 1.2 6–10	1..70	0.167
**Weight (kg)**	♂	22.27 ± 2.94 18–28.1	26.98 ± 4.60 17.6–45.7	32.17 ± 5.2 20.1–47.8	40.71 ± 8.56 24–72.6	264.14	<0.001 *
	♀	22.21 ± 2.51 19–29	27.05 ± 4.98 17–48.9	33.96 ± 6.46 21.6–54	41.63 ± 9.24 26.9–69.8	188.39	<0.001 *
**Height (cm)**	♂	125.77 ± 7.37 112.7–139.5	128.01 ± 8.49 107.3–155	130.42 ± 8.05 107.5–154	133.79 ± 8.36 108–153	22.43	<0.001 *
	♀	126.77 ± 6.41 118–149	128.03 ± 8.74 106–155	131.18 ± 8.9 111–153.4	133.09 ± 9.38 114.2–163.7	11.83	<0.001 *
**BMI (kg·m^−2^)**	♂	13.95 ± 0.81 9.88–14.95	16.34 ± 1.01 14.26–19.10	18.77 ± 0.99 17.12–21.82	22..47 ± 2.5 18.79–32.41	844.36	<0.001 *
	♀	13.79 ± 0.64 12.36–14.97	16.34 ± 1.14 13.92–19.29	19.51 ± 1.3 17.32–23	23.27 ± 2.25 19.48–29.49	828.62	<0.001 *
**Tricipital fold (mm)**	♂	7.57 ± 2.18 4–15	9.68 ± 2.95 4–20	13.74 ± 3.80 6–24	19.36 ± 4.91 8–39	320.99	<0.001 *
	♀	7.28 ± 2.04 4.5–11.8	8.99 ± 3.04 4–31.5	14.77 ± 4.3 6.5–28	20.42 ± 5 7.5–35	306.16	<0.001 *
**Subscap fold (mm)**	♂	3.66 ± 0.91 2–6.5	4.81 ± 1.44 2–11.5	7.20 ± 2.58 4–18	12.48 ± 5.15 4–29.5	310.33	<0.001 *
	♀	3.65 ± 0.97 2–6	4.69 ± 1.96 2–29.5	8.18 ± 3.48 3–25	13.9 ± 5.5 4–30	264.12	<0.001 *
**Add pleats (mm)**	♂	11.23 ± 2.71 7–21.5	14.49 ± 3.87 7–31.5	20.92 ± 5.70 10.5–39	31.84 ± 9.07 12–58	405.01	<0.001 *
	♀	10.93 ± 2.71 6.5–17.8	13.68 ± 4.66 6.5–61	22.94 ± 7.16 9.5–52	34.13 ± 9.97 11.5–64	328.70	<0.001 *
**WC (cm)**	♂	51.75 ± 2.44 49–56.5	55.74 ± 3.48 43–63.4	61.22 ± 4.14 52.50–72.90	70.68 ± 7.32 55.10–88.80	174.52	<0.001 *
	♀	50.53 ± 2.18 47.5–54.5	55 ± 4.64 45.3–86.7	62.26 ± 5.36 51–77.8	69.14 ± 9.44 34.7–87	83.97	<0.001 *
**Fat mass** **(%)**	♂	10.82 ± 2.71 6.38–20.62	14.04 ± 3.67 6.38–28.48	19.85 ± 4.78 10.12–32.14	28.06 ± 6.37 11.67–47.01	419.33	<0.001 *
	♀	10.39 ± 2.76 5.6–17.06	13.01 ± 4.07 5.6–43.01	20.61 ± 5.12 8.96–38.09	27.92 ± 6.04 11.08–44.64	327.53	<0.001 *

Note: Gender (masculine ♂ and feminine ♀); Mean and Standard Deviation (x ± SD); Minimum and Maximum (min.–max.); Abbreviations: BMI—Body mass index; WC—Waist perimeter; * Statistically significant for *p* ≤ 0.05.

**Table 4:** Multiple comparisons between BMI categories in the age variable for both genders.

Variable	BMI Categories			Difference in Averages	*p*	CI 95%	
						Min.	Max.
**Age** ♂ ♀	normal weight	vs.	low weight	−0.11	0.567	−0.50	0.27
	normal weight	vs.	excess weight	0.01	0.916	−0.21	0.24
	excess weight	vs.	low weight	−0.12	0.556	−0.54	0.29
	obesity	vs.	low weight	0.07	0.734	−0.34	0.49
	obesity	vs.	normal weight	0.18	0.104	−0.04	0.41
	obesity	vs.	excess weight	0.20	0.155	−0.07	0.47
	normal weight	vs.	low weight	−0.08	0.763	−0.58	0.43
	normal weight	vs.	excess weight	−0.23	0.032 *	−0.44	−0.02
	excess weight	vs.	low weight	0.15	0.577	−0.38	0.68
	obesity	vs.	low weight	0.08	0.785	−0.48	0.64
	obesity	vs.	normal weight	0.16	0.281	−0.13	0.44
	obesity	vs.	excess weight	−0.07	0.657	−0.39	0.25

* Statistically significant for *p* ≤ 0.05. Abbreviations: CI—Confidence interval; BMI—Body mass index.

**Table 5:** Multiple comparisons between BMI categories in the body composition variables under study, in males (*n* = 792).

Variable	BMI Categories			Difference in Averages	*p*	Confidence Interval (IC 95%)	
						Min.–Max.	
**BMI** **(kg·m^−2^)**	normal weight	vs.	low weight	2.38	<0.001 *	1.93	2.83
	normal weight	vs.	excess weight	−2.43	<0.001 *	−2.69	−2.17
	excess weight	vs.	low weight	4.81	<0.001 *	4.33	5.30
	obesity	vs.	low weight	8.52	<0.001 *	8.03	9.00
	obesity	vs.	normal weight	6.13	<0.001 *	5.87	6.39
	obesity	vs.	excess weight	3.70	<0.001 *	3.39	4.02
**Fat mass** **(%)**	normal weight	vs.	low weight	3.22	<0.001 *	1.80	4.64
	normal weight	vs.	excess weight	−5.81	<0.001 *	−6.65	−4.98
	excess weight	vs.	low weight	9.03	<0.001 *	7.50	10.57
	obesity	vs.	low weight	17.24	<0.001 *	15.71	18.77
	obesity	vs.	normal weight	14.02	<0.001 *	13.20	14.84
	obesity	vs.	excess weight	8.20	<0.001 *	7.21	9.21
**Waist** **Perimeter** **(cm)**	normal weight	vs.	low weight	3.99	0.014 *	0.83	7.15
	normal weight	vs.	excess weight	−5.48	<0.001 *	−6.86	−4.10
	excess weight	vs.	low weight	9.47	<0.001 *	6.17	12.77
	obesity	vs.	low weight	18.92	<0.001 *	15.65	22.20
	obesity	vs.	normal weight	14.94	<0.001 *	13.61	16.26
	obesity	vs.	excess weight	9.46	<0.001 *	7.83	11.09

* Statistically significant for *p* ≤ 0.001.

**Table 6:** Multiple comparisons between BMI categories in the body composition variables under study, in females (*n* = 772).

Variable	BMI Categories			Difference in Averages	*p*	Confidence Interval (95%) Min.–Max.	
**BMI** **(k/m^2^)**	normal weight	vs.	low weight	2.55	<0.001 *	1.99	3.10
	normal weight	vs.	excess weight	−3.18	<0.001 *	−3.40	−2.95
	excess weight	vs.	low weight	5.72	<0.001 *	5.14	6.30
	obesity	vs.	low weight	9.48	<0.001 *	8.87	10.10
	obesity	vs.	normal weight	6.93	<0.001 *	6.62	7.25
	obesity	vs.	excess weight	3.76	<0.001 *	3.41	4.11
**Fat mass** **(%)**	normal weight	vs.	low weight	2.62	0.007 *	0.71	4.52
	normal weight	vs.	excess weight	−7.60	<0.001 *	−8.38	−6.82
	excess weight	vs.	low weight	10.22	<0.001 *	8.24	12.20
	obesity	vs.	low weight	17.53	<0.001 *	15.42	19.64
	obesity	vs.	normal weight	14.91	<0.001 *	13.84	15.98
	obesity	vs.	excess weight	7.31	<0.001 *	6.11	8.50
**Waist** **Perimeter** **(cm)**	normal weight	vs.	low weight	4.47	0.015 *	0.88	8.07
	normal weight	vs.	excess weight	−7.26	<0.001 *	−8.87	−5.64
	excess weight	vs.	low weight	11.73	<0.001 *	7.97	15.49
	obesity	vs.	low weight	18.61	<0.001 *	14.68	22.53
	obesity	vs.	normal weight	14.14	<0.001 *	12.15	16.12
	obesity	vs.	excess weight	6.88	<0.001 *	4.62	9.14

* Statistically significant for *p* ≤ 0.001.

## Data Availability

The authors can be contacted by e-mail to consult them.
